# A 2D Ba_2_N Electride for Transition Metal-Free N_2_ Dissociation under Mild Conditions

**DOI:** 10.1021/jacs.3c09362

**Published:** 2023-10-06

**Authors:** Zhujun Zhang, Yihao Jiang, Jiang Li, Masayoshi Miyazaki, Masaaki Kitano, Hideo Hosono

**Affiliations:** † MDX Research Center for Element Strategy, International Research Frontiers Initiative, Tokyo Institute of Technology, Midori-ku, Yokohama 226-8503, Japan; ‡ WPI-MANA, National Institute for Materials Science, Namiki, Tsukuba, Ibaraki 305-0044, Japan

## Abstract

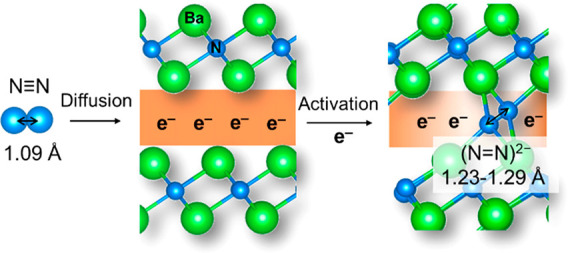

N_2_ activation is a key step in the industrial synthesis of ammonia and other high-value-added N-containing chemicals, and typically is heavily reliant on transition metal (TM) sites as active centers to reduce the large activation energy barrier for N_2_ dissociation. In the present work, we report that a 2D electride of Ba_2_N with anionic electrons in the interlayer spacings works efficiently for TM-free N_2_ dissociation under mild conditions. The interlayer electrons significantly boost N_2_ dissociation with a very small activation energy of 35 kJ mol^–1^, as confirmed by the N_2_ isotopic exchange reaction. The reaction of anionic electrons with N_2_ molecules stabilizes (N_2_)^2–^ anions, the so-called diazenide, in the large interlayer space (∼4.5 Å) sandwiched by 2 cationic slabs of Ba_2_N as the main intermediate.

Dinitrogen activation is a key step in the industrial synthesis of ammonia and many other high-value-added functional N-containing chemicals.^1−3^ However, the high thermodynamic stability of N≡N triple bonds with kinetic inertness (945 kJ mol^–1^) makes dinitrogen activation a significant challenge. Transition metal (TM)-based solid catalysts or complexes are generally necessary for N_2_ activation to reduce the high activation energy barrier.^3,4^ For example, N_2_ activation for ammonia synthesis is well-known to occur on the surface of TMs such as Fe and Ru under high pressures and temperatures.^3,5^ The development of efficient supports or promoters that enhance electron transfer to the TM sites to facilitate N≡N bond weakening through metal-to-N_2_ π-back-donation^6,7^ has been the common approach to decrease the N_2_ activation barrier. For instance, a 12CaO·7Al_2_O_3_ (C12A7:e^–^) electride with a very low work function (2.4 eV) can promote electron transfer to a Ru catalyst, and thus boost N_2_ dissociation on the Ru surface with relatively low activation energy.^8,9^ Although these new strategies can effectively decrease the N_2_ activation energy barrier, TMs are still irreplaceable as active centers in most cases. The development of methods for TM-free N_2_ activation under mild conditions has been a long-standing target due to the potential to realize an environmentally friendly alternative process for N_2_-activation related to the industrial synthesis of chemicals.^10,11^

Alkaline earth metal subnitrides, denoted as *A*e_2_N (*A*e = Ba, Sr, and Ca), are 2D electride materials with anionic electrons sandwiched by cationic slabs of [*A*e_2_N]^2+^ with large interlayer spaces (3.9–4.5 Å).^12−18^ The low work function of *A*e_2_N (<3.0 eV along the (100) direction)^14^ endows them with high electron transport ability, so that they have high potential as catalyst supports to obtain negatively charged TM catalysts for the activation of adsorbed molecules.^11,19−23^ However, the direct activation of molecules such as N_2_ by electride materials in the absence of TM sites has not been reported to date.

In the present work, we report that a 2D Ba_2_N electride works as an efficient catalyst for TM-free N_2_ dissociation with a very small activation energy of 35 kJ mol^–1^, as confirmed by the N_2_ isotopic exchange reaction (N_2_–IER). The (N_2_)^2–^ anion is formed in the large interlayer space of Ba_2_N (∼4.5 Å) by the reaction of the interlayer electron with N_2_, which is confirmed by Raman spectroscopy and density functional theory (DFT) calculations.

The alkaline earth metal subnitride of Ba_2_N was easily prepared by heating pure Ba metal in a N_2_ flow under ambient pressure at elevated temperatures (see Supporting Information). The X-ray diffraction (XRD) pattern for Ba_2_N synthesized at 700 °C is well assigned to a calculated Ba_2_N phase, and the phase crystallizes with a strong (001) orientation (Figure 1a and Figure S1); i.e., the prepared Ba_2_N crystal was oriented along the plane perpendicular to the *c*-axis. This Ba_2_N phase began to form at temperatures as low as 300 °C and further crystallized at higher temperatures over 800 °C without the formation of other barium nitride phases (Figure S1), which suggests that the barium subnitride is the most stable phase in the Ba–N system under ambient N_2_ flow. The microstructure of the as-prepared Ba_2_N powder observed by using scanning electron microscopy (SEM) revealed a typical layered structure (Figure 1b). The sample was a black color due to the metallic nature that originates from itinerant anionic electrons. A single phase of Sr_2_N powder can be prepared by the same synthesis procedure with only Ba replaced by Sr metal (Figure S2). In contrast to Ba_2_N and Sr_2_N, Ca_2_N could not be obtained by the direct nitridation of Ca metal under the same synthesis conditions; instead, Ca_3_N_2_ with a deep red color was obtained (Figure S3), which indicates that Ca_3_N_2_ is the most stable phase of the calcium nitrides. Ca_2_N powder could be prepared by the reduction of Ca_3_N_2_ with Ca metal at 800 °C^12,19^ (Figure S4).

**Figure 1:**
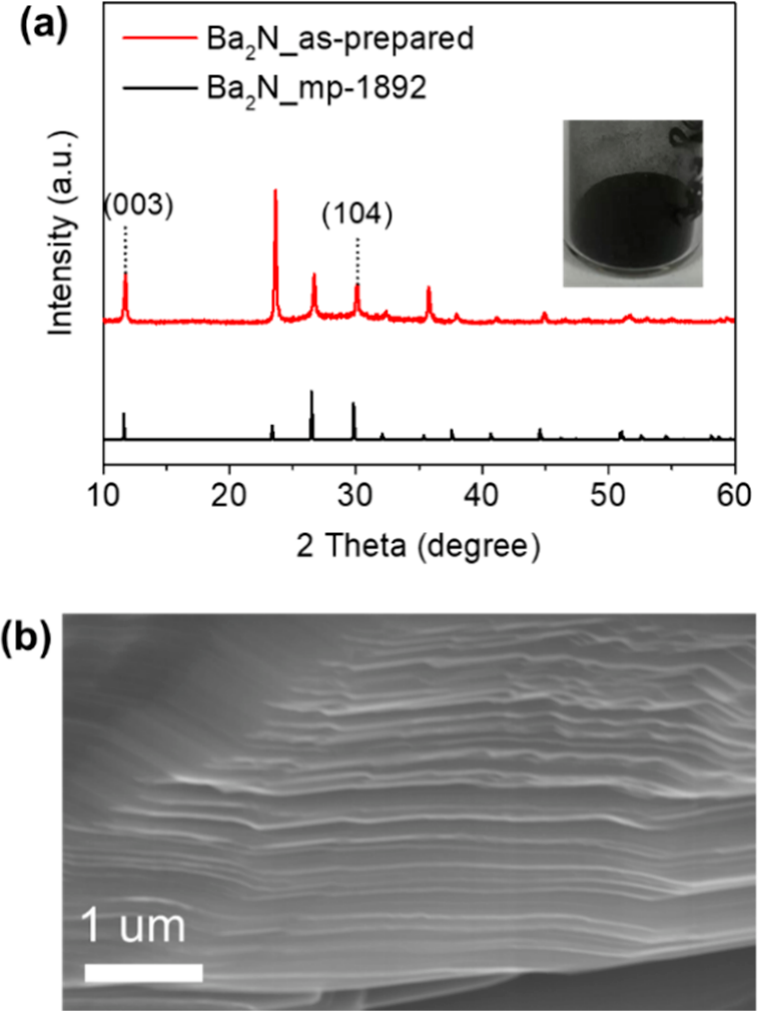
(a) XRD pattern for as-prepared Ba_2_N synthesized at 700 °C. The inset shows the photo of collected black Ba_2_N powder. (b) SEM image of as-prepared Ba_2_N.

As a 2D electride, Ba_2_N is first shown to have high efficiency for N_2_ dissociation as confirmed by N_2_–IER under 4 kPa of ^15^N_2_ and 16 kPa of ^14^N_2_ (Figure 2a). The mass signals at *m*/*z* = 28, 29, and 30 were monitored with the reaction time at various reaction temperatures (Figures S5–S11). Alkaline earth metal nitrides, such as Sr_3_N_2_, Ca_3_N_2_, and Mg_3_N_2_, as nonelectride references, showed no activity for the N_2_–IER, even at temperatures up to 500 °C, which indicates that they cannot dissociate dinitrogen molecules. In contrast, the Ba_2_N alkaline earth metal subnitride functioned as an efficient catalyst for N_2_ dissociation with outstanding activity above 200 °C (0.7 mmol g^–1^ h^–1^) and reached 23.1 mmol g^–1^ h^–1^ at 400 °C, which is far beyond the activity of the conventional Ru/MgO catalyst. Sr_2_N also acted efficiently for the N_2_–IER above 200 °C (0.6 mmol g^–1^ h^–1^) and reached 9.5 mmol g^–1^ h^–1^ at 400 °C. In contrast to Ba_2_N and Sr_2_N, Ca_2_N exhibited much lower activity for the N_2_–IER. Isotopic N_2_ exchange over Ca_2_N began above 375 °C (0.3 mmol g^–1^ h^–1^) and reached 4.1 mmol g^–1^ h^–1^ at 500 °C. The activation energy (*E*_a_) of Ba_2_N and Sr_2_N for the N_2_–IER was as low as 34.3 and 38.8 kJ mol^–1^, respectively, which is much lower than that of Ca_2_N (68.3 kJ mol^–1^), the conventional Ru/MgO catalyst (108.3 kJ mol^–1^) (Figure 2b), and even lower than that (58 kJ mol^–1^) of the Ru/C12A7:e^–^ catalyst.^8^ These results demonstrate that 2D alkaline earth metal subnitrides of Ba_2_N and Sr_2_N can dissociate N_2_ with a very small energy barrier in the absence of TM sites.

**Figure 2:**
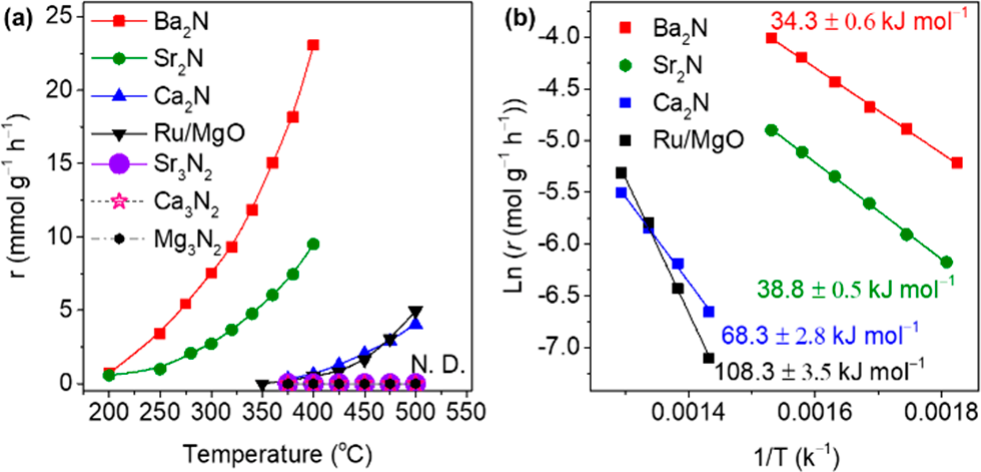
(a) N_2_–IER rate for *A*e_2_N, *A*e_3_N_2_ and the conventional Ru/MgO catalyst at various temperatures. (b) Corresponding Arrhenius plots for *A*e_2_N and Ru/MgO.

To clarify the N_2_ activation dynamics with the *A*e_2_N electrides, pure ^15^N_2_ (20 kPa) was used to react with these electrides at 400 °C, and the mass signal at *m*/*z* = 29 (^14^N^15^N) was monitored with the reaction time (Figure 3a and Figures S12–S14). For Ba_2_N and Sr_2_N, the *m*/*z* = 29 signal increased quickly with reaction time, which suggests their lattice nitrogen species attended the nitrogen dissociation process and exchange with molecular nitrogen of ^15^N_2_. The mass signal intensity at *m*/*z* = 29 for Ca_2_N did not increase with reaction time under the same reaction conditions, which suggests that Ca_2_N follows different N_2_ dissociation pathways than Ba_2_N and Sr_2_N. The rates for *m*/*z* = 29 formation under isotopic N_2_–IER conditions and pure ^15^N_2_ treatment conditions over *A*e_2_N electrides are summarized in Figure 3b. The results clarify that the N_2_ dissociation activity of both Ba_2_N and Sr_2_N is mainly induced by the exchange of lattice nitrogen species during the N_2_ dissociation process, while Ca_2_N only follows the direct N_2_ dissociation process.

**Figure 3:**
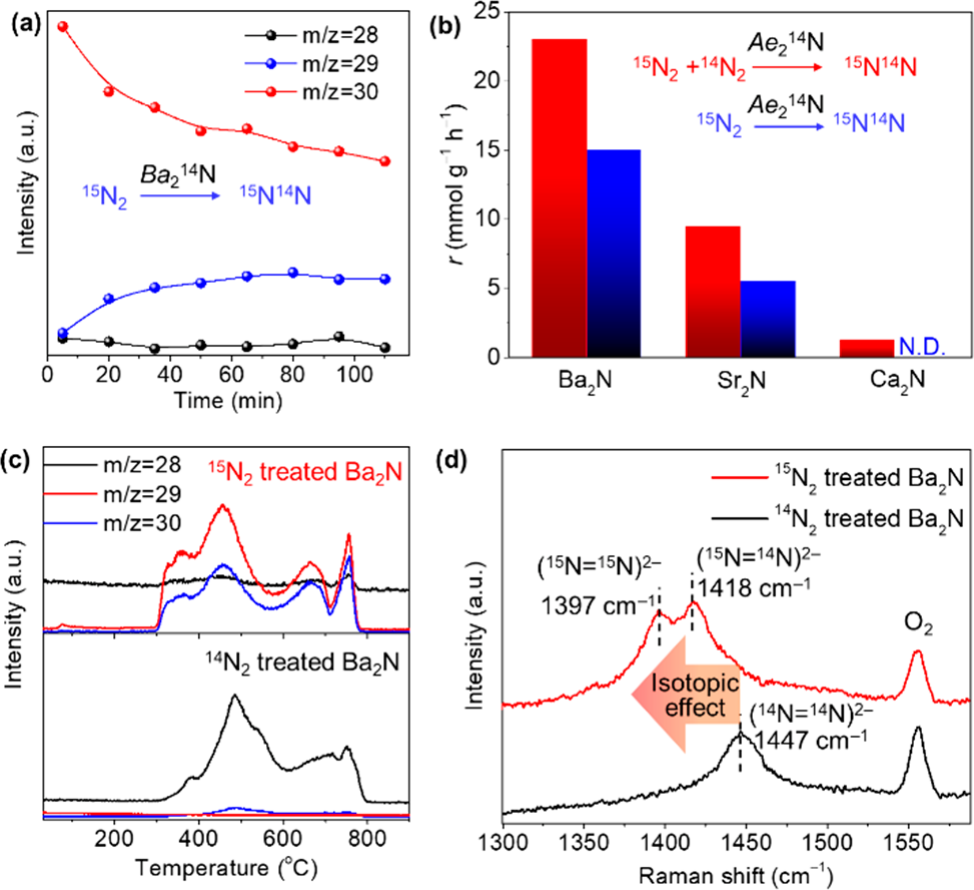
(a) Monitoring of mass signal intensities at *m*/*z* = 28, 29, and 30 as a function of the reaction time when Ba_2_N reacts with pure ^15^N_2_ (20 kPa) at 400 °C. (b) Comparison of the N_2_–IER rates measured under different gas atmospheres at 400 °C. Red columns: 16 kPa ^14^N_2_ + 4 kPa ^15^N_2_; Blue columns: 20 kPa ^15^N_2_. (c) TPD of N_2_ desorption (c) and Raman spectra (d) for Ba_2_N collected by 20 kPa of pure ^14^N_2_ (bottom) or ^15^N_2_ (top) treatment at 400 °C for 2 h.

Temperature-programmed desorption (TPD) of N_2_ from Ba_2_N collected after treatment with pure ^14^N_2_ or ^15^N_2_ (20 kPa) was performed (Figure 3c) to determine the intermediates produced on/in Ba_2_N during N_2_ dissociation. When the as-prepared Ba_2_N was pretreated in pure ^14^N_2_, N_2_ desorption peaks at low temperatures (290–570 °C) were observed. This is different from Ca_2_N, which has only N_2_ desorption from lattice N^3**–**^ ions at over 600 °C (Figure S15). After Ba_2_N was heat treated in ^15^N_2_, the *m*/*z* = 28 (^14^N_2_) signal intensity almost disappeared, and instead, strong signals at *m*/*z* = 30 and 29 with the same peak shape emerged in the same temperature range. The fraction of exchanged ^14^N with ^15^N is estimated to be 14.4%. Similar results were also observed for Sr_2_N, but not for Ca_2_N (Figures S15 and S16). Raman analysis was performed to identify these activated nitrogen species (Figure 3d and Figure S17). The feature signals that appeared at 1554 cm^**–**1^ for all of the tested samples were due to atmospheric O_2_. The sample collected after pure ^14^N_2_ treatment showed a main signal centered at 1447 cm^**–**1^. After the ^15^N_2_ treatment, the signal was split into two peaks at 1397 and 1418 cm^–1^, which agreed well with the isotopic effect induced by the change of ^14^N_2_ to ^15^N_2_ and ^15^N^14^N. These signals can be attributed to stretching vibrations of (N_2_)^2**–**^ anions, so-called diazenide, as identified in high-pressure stabilized inorganic materials and organic complexes.^1,24−27^ Moreover, the Raman signal for (N_2_)^2 **–**^ anions almost disappeared when Ba_2_N was further heated in He at 600 °C (Figure S18). Therefore, N_2_ desorption in the low-temperature range (290–570 °C) is confirmed to result from the release of (N_2_)^2**–**^ anions that are formed as intermediates of N_2_ dissociation on/in Ba_2_N.

DFT calculations were performed to determine the structure of the incorporated (N_2_)^2**–**^ anions in Ba_2_N (see Supporting Information, Figure S19 and Table S1). When pure Ba_2_N reacts with N_2_ molecules (Ba_24_N_12_+3N_2_) (Figure 4), (N_2_)^2**–**^ anions are captured in the interlayers of Ba_2_N through reaction with the anionic electrons. The stabilization of (N_2_)^2**–**^ anions in Ba_2_N proceeds with the formation energy of **–**2.92 eV, which indicates that N_2_ molecules can be readily incorporated into the interlayers of Ba_2_N by binding with the Ba ions present in the upper and lower layers. The bond length for the stabilized dinitrogen was 1.23–1.29 Å (Table S2), which is much longer than that for free N_2_ (1.09 Å) but similar to the (N_2_)^2**–**^ (∼1.20–1.35 Å).^1^ The corresponding N_2_ vibration was calculated to be 1424–1448 cm^**–**1^ (Table S2), which is very close to the Raman analysis result (∼1446 cm^**–**1^). The electron localization function calculation for Ba_2_N with and without (N_2_)^2**–**^ anions is shown in Figure 4 (right side), and the Bader charge analysis of an (N_2_)^2**–**^ anion in the model of Ba_24_N_12_+3N_2_ is shown in Figure S20. The high-density anionic electron layers are located in the interlayer spaces of pure Ba_2_N. After the formation of an (N_2_)^2**–**^ anion within an interlayer, the electron concentration in the interlayer was distinctly decreased compared to that for pure Ba_2_N. This is because the N_2_ molecules were activated as (N_2_)^2**–**^ ions by accepting electrons from the interlayer, and the resultant (N_2_)^2**–**^ ions are stabilized electrostatically by coordination with Ba^2+^ ions. The electron concentration of Ba_2_N (0.002 mol g^–1^) was evaluated by a iodometric titration (Table S3),^28^ which suggests the molar ratio for Ba_2_N:e^**–**^ is 1.0:0.6. This ratio is significantly lower than the theoretical ratio of 1.0:1.0 for Ba_2_N, which confirms that the (N_2_)^2 **–**^ anions were formed in the interlayers through the consumption of a portion of the electrons.

**Figure 4:**
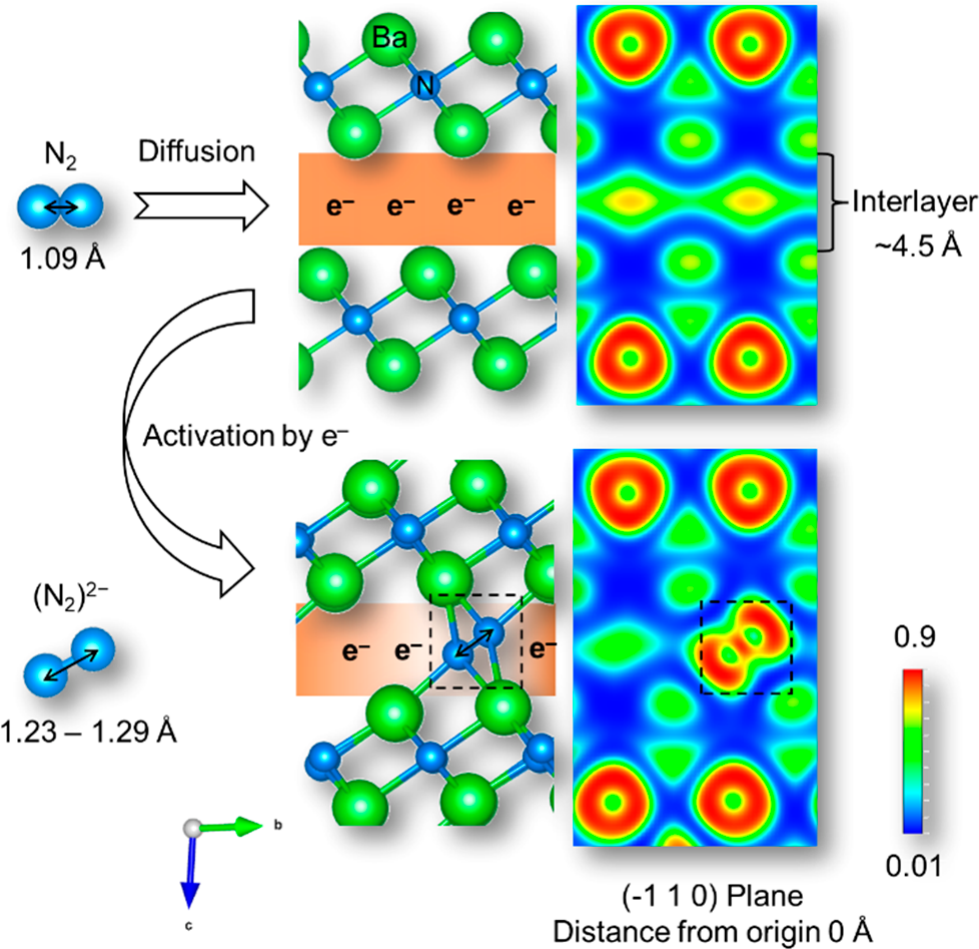
Schematic illustration of (N_2_)^2–^ anion formation in the interlayers of Ba_2_N as demonstrated by DFT calculations.

Ca_2_N shows a significantly weaker ability for N_2_ activation than Ba_2_N and Sr_2_N. According to the DFT calculation results, the stabilized (N_2_)^2 **–**^ anions are likely to be dissociated at the interlayer space of Ba_2_N (Figure S21). The work functions of Ba_2_N and Ca_2_N for the (104) plane are calculated as 2.05 and 2.55 eV, respectively. As the work function of Ba_2_N is close to or slightly lower than the lowest unoccupied molecular orbital (LUMO) energy level of the N_2_ molecule (∼2.1 eV), electron donation from the Ba_2_N to N_2_ molecule, therefore, easily occurs (Figure S22). However, the work function of Ca_2_N is a little bit larger, making it difficult to donate its interlayer electron to the N_2_ molecule. This is why Ca_2_N shows a much larger activation energy for N_2_–IER compared to that of Ba_2_N.

In conclusion, we have demonstrated that an easily obtained 2D Ba_2_N electride works efficiently for TM-free N_2_ dissociation with a very small activation energy of 35 kJ mol^–1^. Isotopically labeled TPD and Raman analyses, and DFT calculation results clearly confirmed the formation of the (N_2_)^2–^ anion, so-called diazenide, in the interlayer space of Ba_2_N as the main intermediate. This study thus demonstrated a new way to realize TM-free N_2_ activation under mild conditions.

## Supporting Information Available

The experimental section, which includes the synthesis of *A*e_2_N, N_2_–IER measurement methods, TPD of N_2_, Raman observations, and DFT calculation methods can be found in the Supporting Information. (PDF)
