# Supplementary material for: A 2D Ba2N Electride for Transition Metal-Free N2 Dissociation under Mild Conditions
Source: J Am Chem Soc. 2023 Oct 6;145(45):24482–5. doi: 10.1021/jacs.3c09362 (PMC10655079; doi:10.1021/jacs.3c09362)
Supplement: Supplementary file 1 — ja3c09362_si_001.pdf [file ja3c09362_si_001.pdf]

Supplementary Information

A 2D Ba_2N Electride for Transition Metal-free N_2 Dissociation under Mild Conditions

Zhujun Zhang,[*,§,†] Yihao Jiang,[§,†] Jiang Li,[†] Masayoshi Miyazaki,[†] Masaaki Kitano,[*,†] Hideo Hosono[*,§,†,‡]

[†]MDX Research Center for Element Strategy, International Research Frontiers Initiative, Tokyo Institute of Technology, Midori-ku, Yokohama 226-8503, Japan;

[‡]WPI-MANA, National Institute for Materials Science, Namiki, Tsukuba, Ibaraki 305-0044, Japan;

[§]These authors contributed equally to this work.

[*]Email: zhangzhujun11@gmail.com; kitano.m.aa@m.titech.ac.jp; hosono.h.aa@m.titech.ac.jp

1. Experimental Section

1.1 Synthesis of Ba_2N and Sr_2N

Ba_2N was synthesized by the direct nitridation of the corresponding Ba metal in a pure N_2 flow under ambient pressure at elevated temperatures. Typically, 1.5 g of granular Ba metal nuggets (99.99%, Aldrich Co.) was set in a molybdenum metal crucible and transferred to a quartz tube reactor in an Ar-filled glove box. Pure N_2 gas (50 mL min^{-1}) was then flowed through this reactor under ambient pressure, heated to the target temperature (300-800 °C), and held for 15 h. The heating rate was kept at 5 °C min^{-1}. After cooling naturally, the metal slag-like product was collected and then ground to a powder in an Ar-filled glove box for further use. Sr_2N was synthesized in the same way as Ba_2N using Sr metal (99.99%, Aldrich Co.) as a precursor.

1.2 Synthesis of Ca_2N

Ca_2N powder was synthesized by the solid-state reaction of Ca_3N_2 powder and Ca metal shot. Dark red Ca_3N_2 powder was prepared by heating Ca metal shot (99.99%, Aldrich Co.) in a pure N_2 flow at 700 °C for 15 h. The as-prepared Ca_3N_2 powder was mixed with Ca metal shot at a molar ratio of 1:1.2 and the mixture was uniaxially pressed into a pellet form under a pressure of ~20 MPa. The pellet was set in a molybdenum foil cell and sealed in an evacuated silica tube. The silica tube was heated at 800 °C for 50 h. The obtained sample was then ground into a dark green powder in an Ar-filled glove box for further use. X-ray diffraction (XRD) patterns for the obtained samples were consistent with the standard diffraction pattern for Ca_2N (Figures. S3 and S4).

1.3 Sample characterization

Powder XRD patterns for the samples were recorded on a diffractometer (D8 Advance, Bruker, Germany) with monochromated Cu Kα radiation (λ = 1.5418 Å) at a voltage of 40 kV and a current of 40 mA. An X-ray transmitting airtight specimen holder with a non-reflective silicon plate was used to avoid oxidation of the sample and X-ray reflection from sources other than the sample during the XRD measurements. Temperature programmed desorption (TPD) experiments were performed on a catalyst analyzer (BELCAT-A, MicrotracBEL, Japan). The sample (~50 mg) was heated from room temperature to 900 °C with a heating rate of 10°C min^{-1} in a stream of pure He gas (>99.99995%) with a flow rate of 30 mL min^{-1}. The products desorbed during the heating process were monitored using mass spectrometry (BELMass, MicrotracBEL, Japan). Raman spectra of the samples were measured with a spectrometer (HR-800, Horiba Jobin Yvon Co. Ltd., Japan). Continuous wave-diode pumped solid-state (CW-DPSS) laser light (λ = 457.4 nm, 50 mW) was focused onto the sample surface through an objective lens, and an energy cutoff filter was used to prevent thermal decomposition induced by laser irradiation. The 2D Ae_2N electride has unpaired electrons; therefore, it is not possible to apply electron paramagnetic resonance (EPR) measurements to determine electron concentration. Therefore, iodometric titration the experiments were used as an effective alternative method to determine the electron concentration of Ba_2N. Field-emission scanning electron microscopy (FE-SEM) observations were conducted on a field-emission scanning electron microscope (JSM–7600F, JEOL).

1.4 N_2 isotopic exchange reaction measurement.

N_2 isotopic exchange reaction measurements of all the samples were performed in a closed U-shaped glass circulation system connected to a quadrupole mass spectrometer (M-101QA-TDM, Canon Anelva Corp.) using He as the carrier gas. A mixture of $^{15}N_2$ (4.0 kPa) and $^{14}N_2$ (16.0 kPa) gases was introduced and kept circulated in the glass circulation system, and the catalyst (0.1 g) set in the system was heated at target temperatures. During the measurement, reaction gas mixture was abstracted from the gas circulation system to a sampling loop and injected to a quadrupole mass spectrometer to obtain time evolution spectra. The peaks at m/z = 28, 29 and 30 were monitored as a function of time. To study the N_2 dissociation mechanism on Ae_2N, pure $^{15}N_2$ (20 kPa) or $^{14}N_2$ (20 kPa) was introduced into the same system and the peaks at m/z = 28, 29 and 30 were monitored as a function of the reaction time at 400°C.

2. DFT calculations

All density functional theory (DFT) calculations were implemented in the Vienna Ab initio Simulation Package (VASP).[1,2] The generalized gradient approximation with the Perdew–Burke–Ernzerhof functional was adopted in the DFT calculations, and the core electrons were described using the projector-augmented wave (PAW) method.[3] A plane wave cutoff energy of 500 eV was used for all calculations. The convergence criteria for energy and force were respectively 1.0×10^{-6} eV and 1.0×10^{-2} eV $Å^{-1}$ for all calculations. The models for Ba_2N with absorbed interlayer N_2 molecule(s) were built by inserting N_2 molecule(s) into the interlayer of a pristine Ba_2N unit cell (Ba_6N_3) or a supercell composed of Ba_2N unit cells. The crystal structures were visualized using the VESTA program.[4] Gamma-centered k-point meshes of 9×9×1, 4×2×1, 4×2×1 and 4×4×1 were used for the pristine Ba_6N_3, $Ba_{36}N_{18}$+N_2 (2×3×1 of unit cells), $Ba_{36}N_{18}$+3N_2 (2×3×1 of unit cells) and $Ba_{24}N_{12}$+3N_2 (2×2×1 of unit cells).

The N_2 adsorption characteristics were first investigated using models for $Ba_{36}N_{18}+N_2$. One N_2 molecule can be inserted into the interlayer of the Ba_2N lattice both horizontally and perpendicularly along the c-axis. There are a total number of 15 types of adsorption structures. It has been observed that the horizontally oriented N_2 molecule interacts with adjacent Ba ions above and below, which results in the formation of a perpendicular adsorption structure. Therefore, it should be noted that the interlayer of the Ba_2N lattice does not allow for horizontal adsorption of N_2 molecules. After relaxation, it has been observed that the 15 types of adsorption structures exhibit a propensity to form three distinct and comparatively stable adsorption structures (Figure S19 and Table S1). The N_2 incorporation energy for Ba_2N is defined as:

$$E_f = E(Ba_{36}N_{18}+N_2) – E(Ba_{36}N_{18}) – E(N_2)$$

where E(X) is the total system energy of model X.

Following the identification of the most stable adsorption structure (M3 in Supplementary Table 1), models were built featuring different N_2 adsorption density ($Ba_{36}N_{18}+N_2$, $Ba_{36}N_{18}+3N_2$ and $Ba_{24}N_{12}+3N_2$), and the vibrations of N_2 molecules in these models were calculated (Table S2). Other lattice ions were fixed when calculating the vibrations of adsorbed N_2 molecules. The electron-localization function (ELF) of the total electron density at z/c =0 on the (–1 1 0) plane was calculated for $Ba_{24}N_{12}+3N_2$ and Ba_6N_3. Bader analysis of the charge density distributions was performed for the $Ba_{24}N_{12}+3N_2$ model.[5]

The (104) plane of Ba_2N is the most stable plane due to its lowest formation energy. As a result, this plane was selected to build the slab models for the subsequent investigation of N_2 dissociation pathways and work function. Supercells with a vacuum layer of 20 Å along the c axis were built in these slab models to avoid the interactions caused by the periodically repeated slab surfaces.

The slab models consist of six layers with the final two layers at the bottom frozen. Work function is defined as the difference between the vacuum level above the relaxed surface and fermi level of various materials. The climbing image nudged elastic band (CI-NEB) method was used for estimation of reaction pathway and energy barrier for each elementary step of the N_2 dissociation and diffusion.[6] The convergence criteria of density matrix and force are respectively 1.0×10^{-5} and 2.0×10^{-2}eV Å^{-1}.

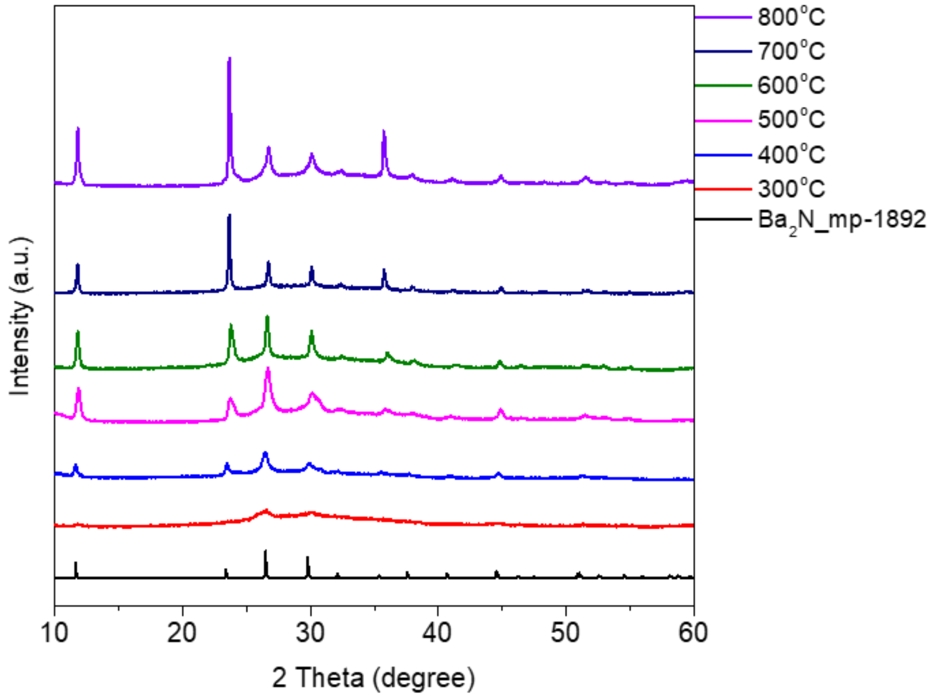

Figure S1. XRD patterns for Ba_2N synthesized at various temperatures.

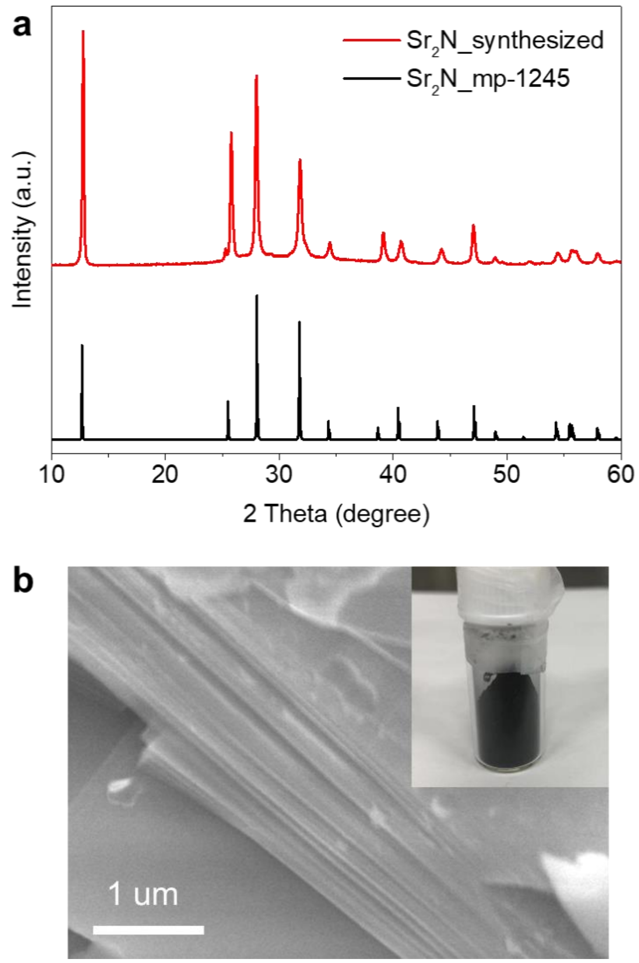

Figure S2. (A) XRD pattern for as-prepared Sr_2N synthesized by directly heating pure Sr shot in a pure N_2 flow under ambient pressure at 700°C for 15 h. (B) SEM image of as-prepared Sr_2N. The inset shows the collected black Sr_2N powder.

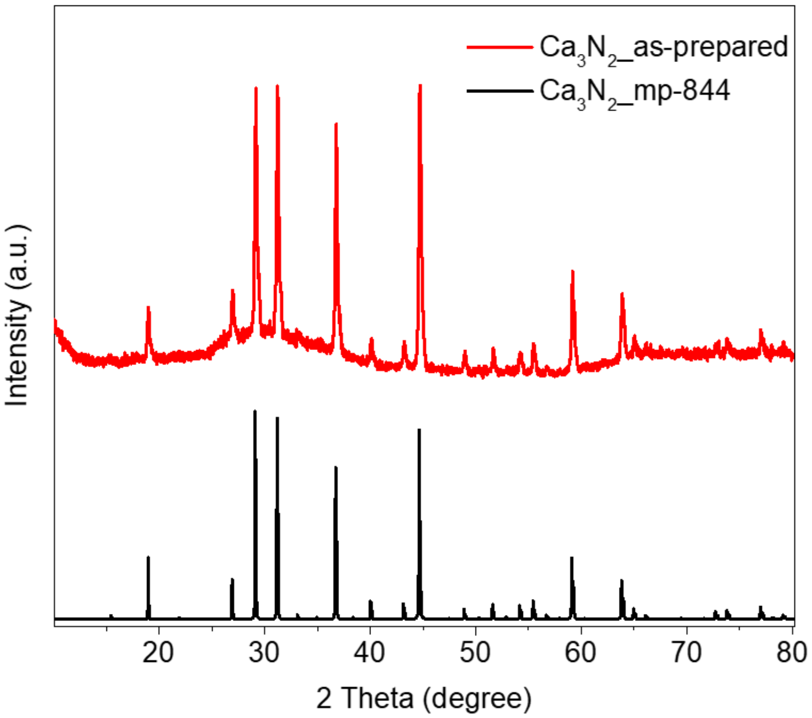

Figure S3. XRD pattern for as-prepared Ca_3N_2 synthesized by directly heating pure Ca shot in a pure N_2 flow under ambient pressure at 700°C for 15 h.

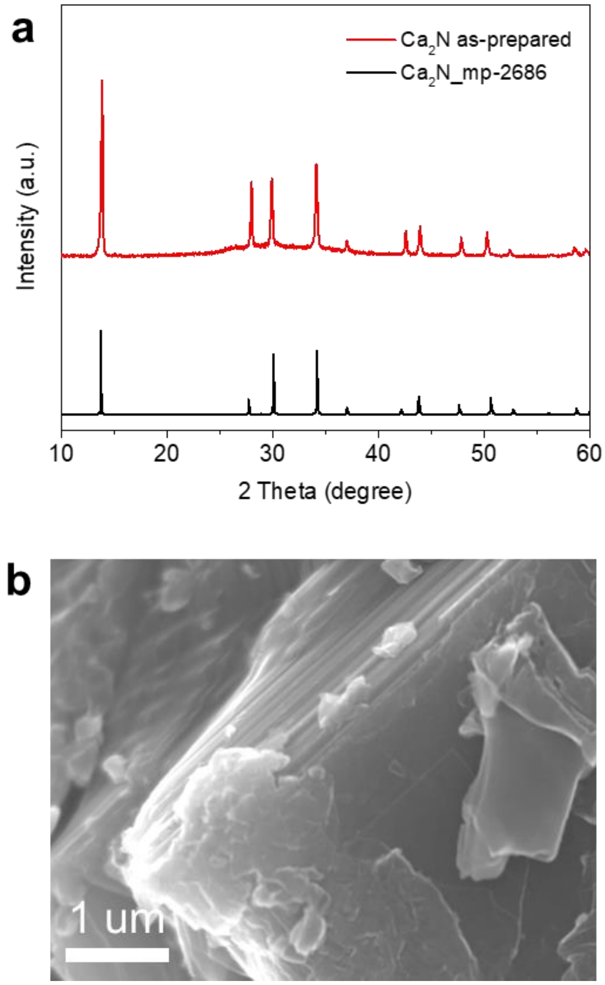

Figure S4. (A) XRD pattern for as-prepared Ca_2N and calculated Ca_2N (Ca_2N_mp-2686). (B) SEM image of as-prepared Ca_2N.

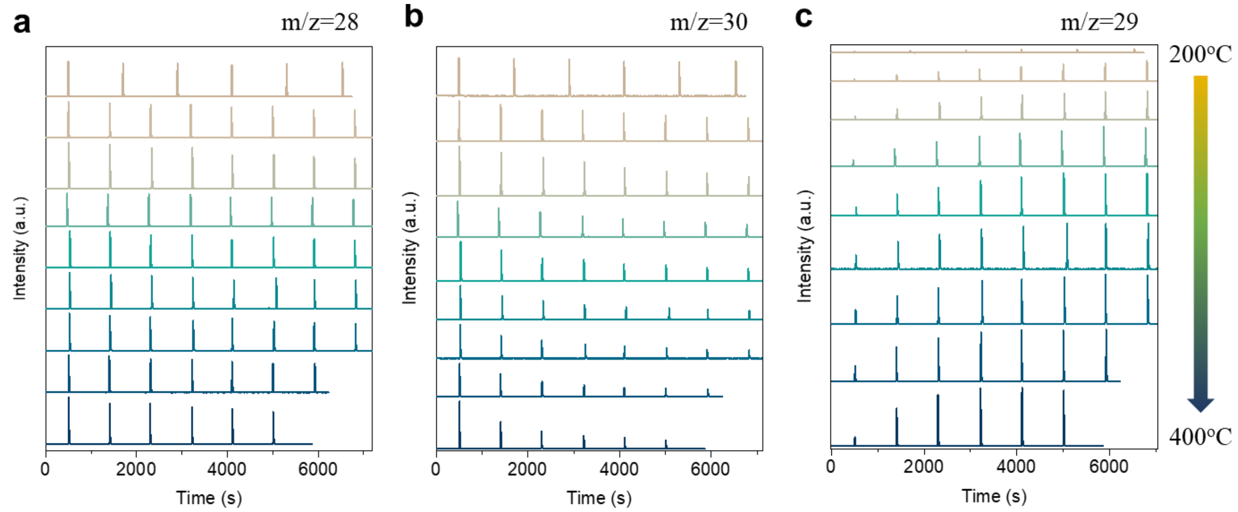

Figure S5. Time evolution of mass spectra peaks at (a) m/z=28, (b) m/z=30, and (c) m/z=29 for isotopic N_2 exchange from $^{15}N_2$ (4 kPa) and $^{14}N_2$ (16 kPa) over Ba_2N at various temperatures (200-400°C). The mass signal intensity of m/z=29 was increased with reaction time again with the decrease of mass signal intensity of m/z=28 and 30. The m/z=29 formation rate was increased with the increase of reaction temperatures.

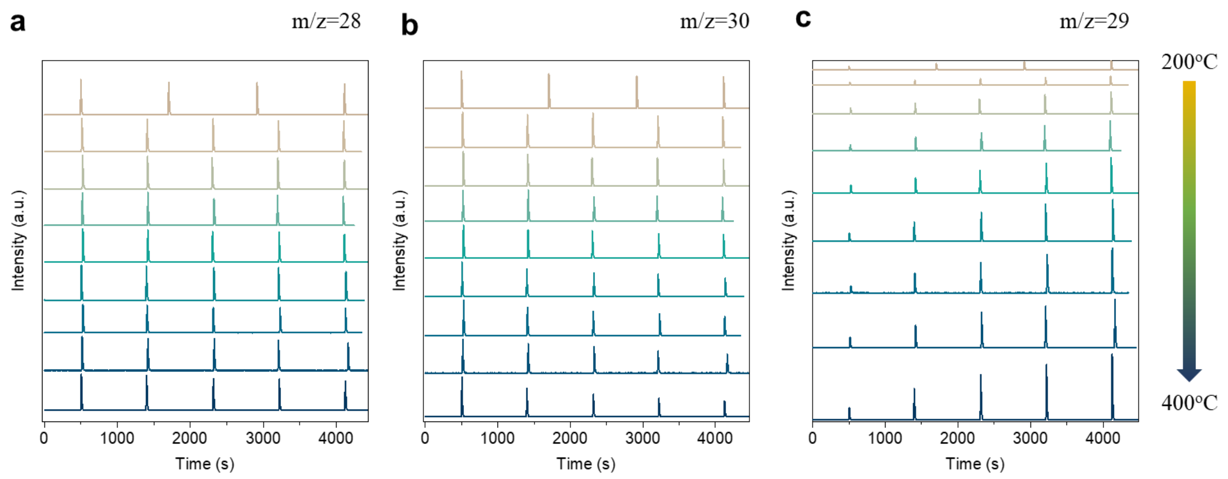

Figure S6. Time evolution of mass spectra peaks at (a) m/z=28, (b) m/z=30, and (c) m/z=29 for isotopic N_2 exchange from $^{15}N_2$ (4 kPa) and $^{14}N_2$ (16 kPa) over Sr_2N at various temperatures (200-400°C). The mass signal intensity of m/z=29 was increased with reaction time again with the decrease of mass signal intensity of m/z=28 and 30. The m/z=29 formation rate was increased with the increase of reaction temperatures.

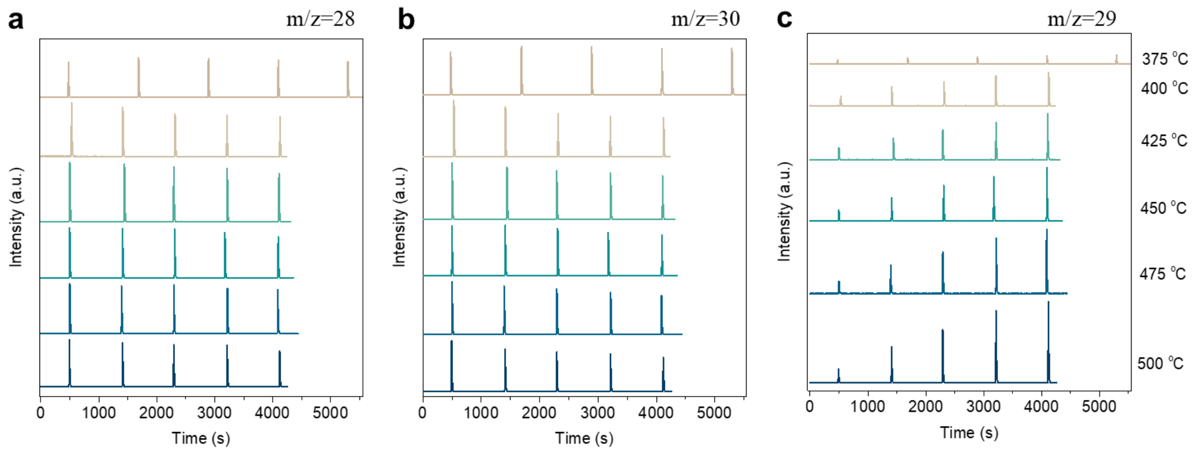

Figure S7. Time evolution of mass spectra peaks at (a) m/z=28, (b) m/z=30, and (c) m/z=29 for isotopic N_2 exchange from $^{15}N_2$ (4 kPa) and $^{14}N_2$ (16 kPa) over Ca_2N at various temperatures (375-500°C). The mass signal intensity of m/z=29 was increased with reaction time again with the decrease of mass signal intensity of m/z=28 and 30. The m/z=29 formation rate was increased with the increase of reaction temperatures.

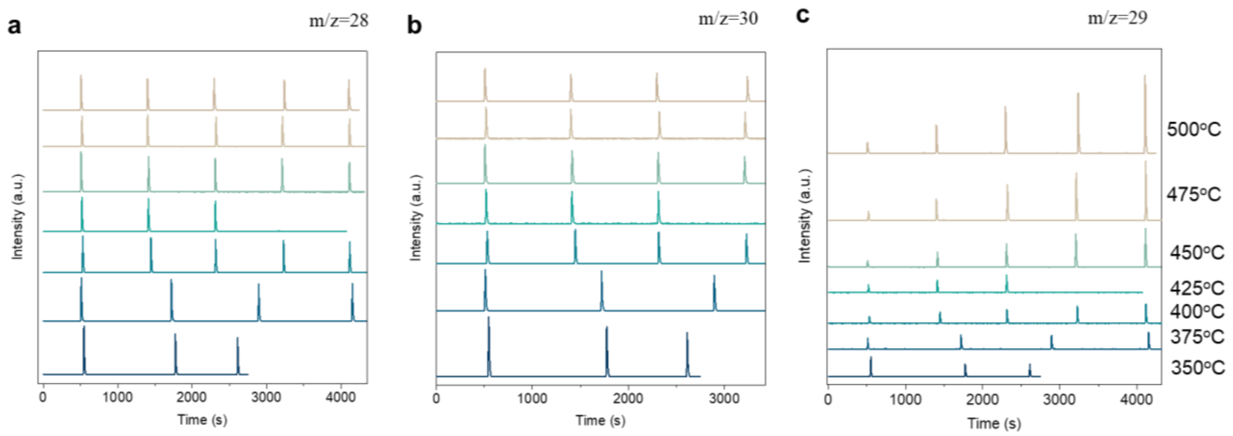

Figure S8. Time evolution of mass spectra peaks at (a) m/z=28, (b) m/z=30, and (c) m/z=29 for isotopic N_2 exchange from $^{15}N_2$ (4 kPa) and $^{14}N_2$ (16 kPa) over Ru/MgO at various temperatures (350-500°C). The mass signal intensity of m/z=29 was increased with reaction time again with the decrease of mass signal intensity of m/z=28 and 30. The m/z=29 formation rate was increased with the increase of reaction temperatures.

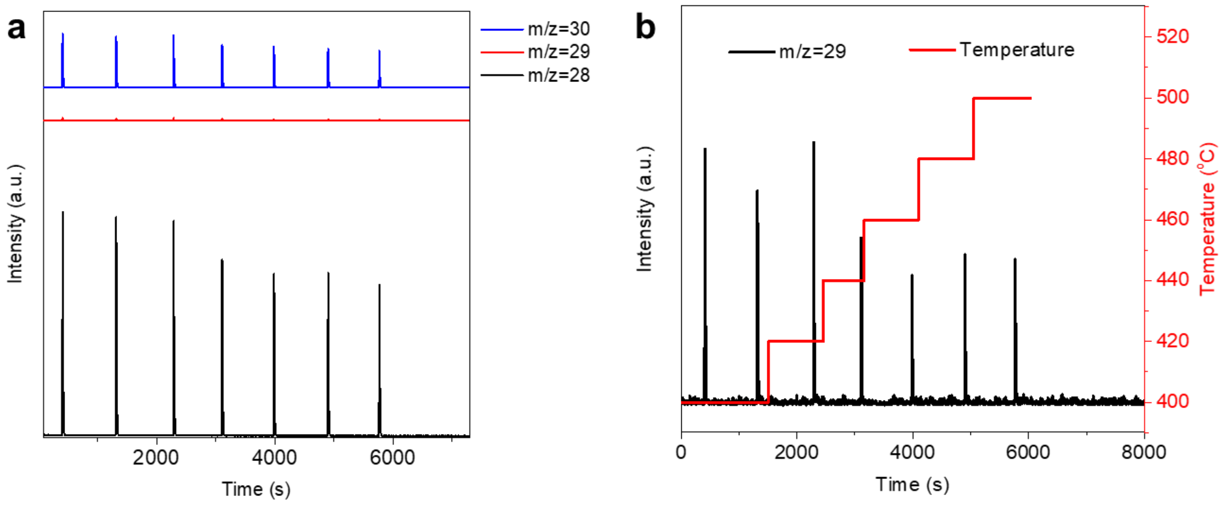

Figure S9. (a) Time evolution of mass spectra peaks at m/z=28, m/z=29, and m/z=30 during isotope N_2 exchange measurement for commercial Sr_3N_2 at various temperatures (400-500 °C). (b) Time evolution of m/z=29 peak shown in panel a and the heating program during the test.

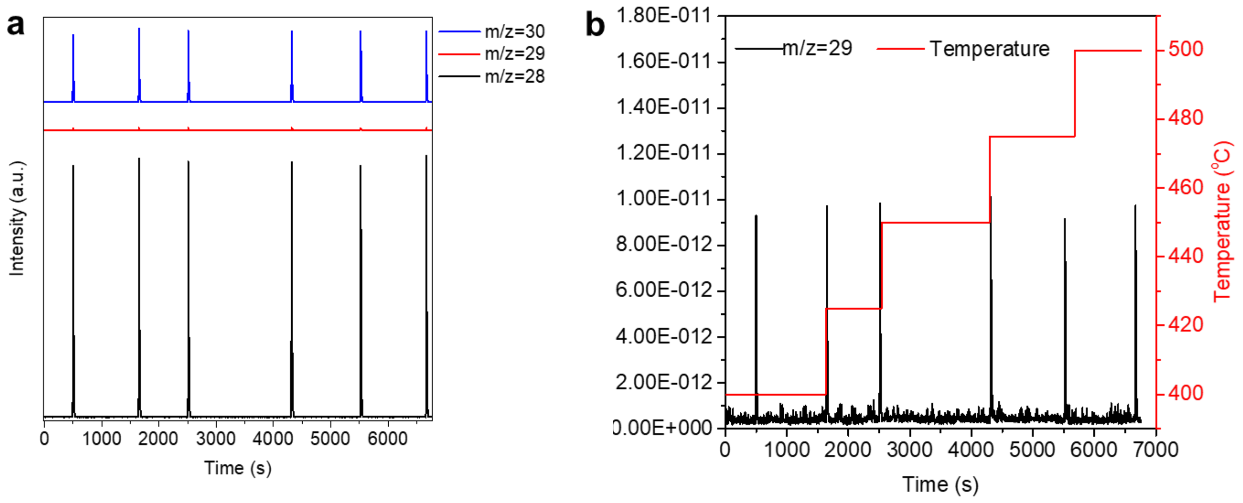

Figure S10. (a) Time evolution of mass spectra peaks at m/z=28, m/z=29, and m/z=30 during isotope N_2 exchange measurement for commercial Ca_3N_2 at various temperatures (400-500°C). (b) Time evolution of mass spectra peak at m/z=29 shown in panel a and the heating program during the test.

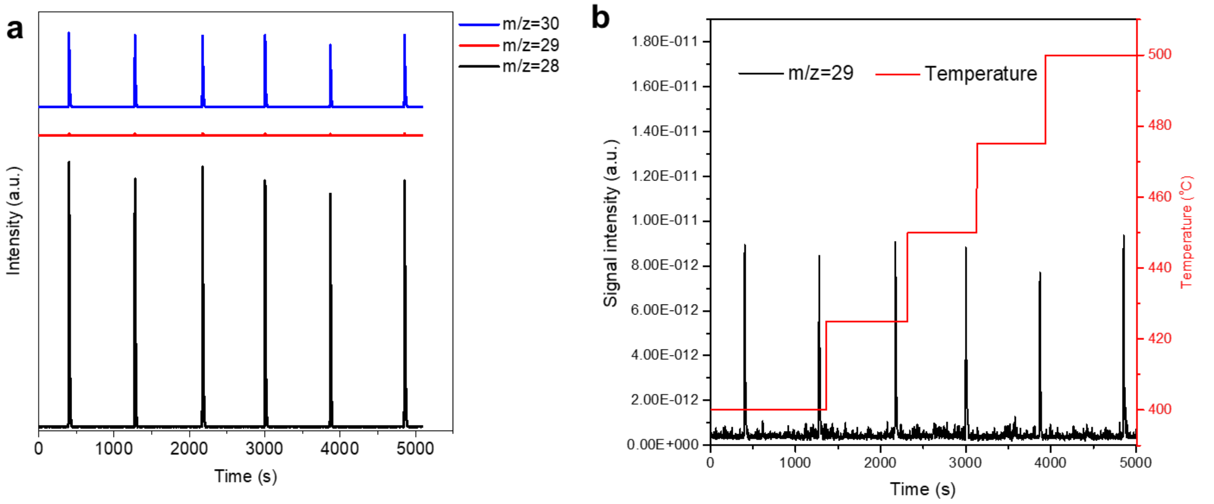

Figure S11. (a) Time evolution of mass spectra peaks at m/z=28, m/z=29, and m/z=30 during isotope N_2 exchange measurement for commercial Mg_3N_2 at various temperatures (400-500°C). (b) Time evolution of mass spectra peak at m/z=29 shown in panel a and the heating program during the test.

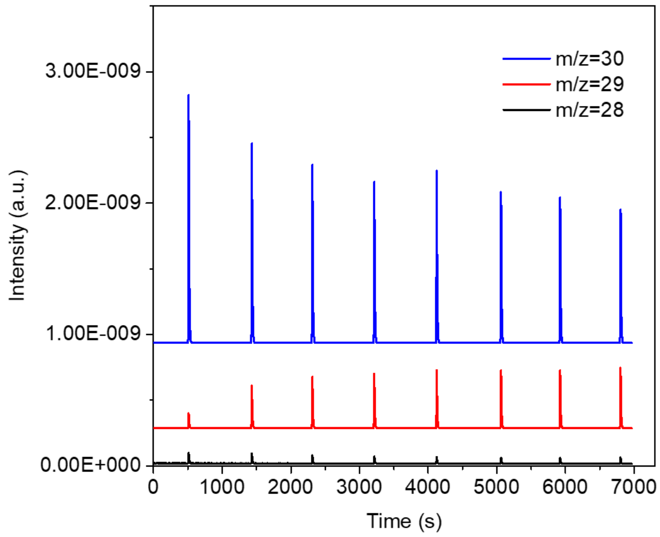

Figure S12. (a) Time evolution of mass spectra peaks at m/z=28, m/z=29, and m/z=30 during pure $^{15}N_2$ (20 kPa) treatment of Ba_2N at 400°C. The mass signal intensity of m/z=29 was increased with reaction time again with the decrease of mass signal intensity of m/z=30 without formation of mass signal m/z=28. This means lattice nitrogen species facilitate the nitrogen dissociation process and exchange with molecular nitrogen ($^{15}N_2$).

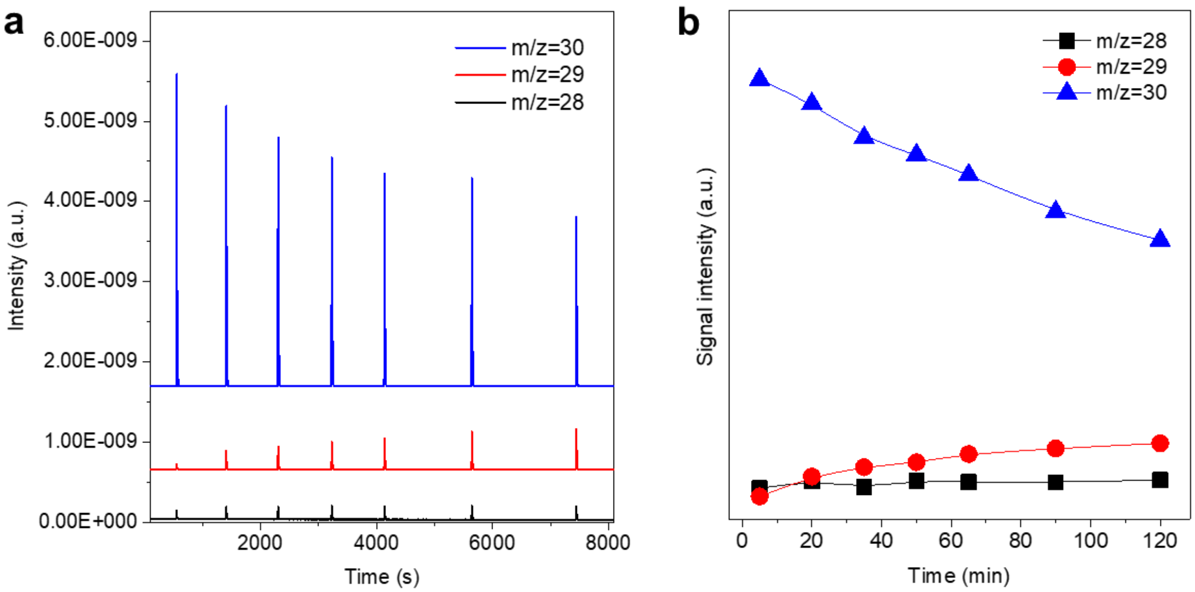

Figure S13. (a) Time evolution of mass spectra peaks at m/z=28, m/z=29, and m/z=30 during pure $^{15}N_2$ (20 kPa) treatment of Sr_2N at 400°C. (b) Intensity of peaks at m/z=28, 29, and 30 monitored as a function of the reaction time in panel a. The mass signal intensity of m/z=29 was increased with reaction time again with the decrease of mass signal intensity of m/z=30 without formation of mass signal m/z=28. This means lattice nitrogen species facilitate the nitrogen dissociation process and exchange with molecular nitrogen ($^{15}N_2$).

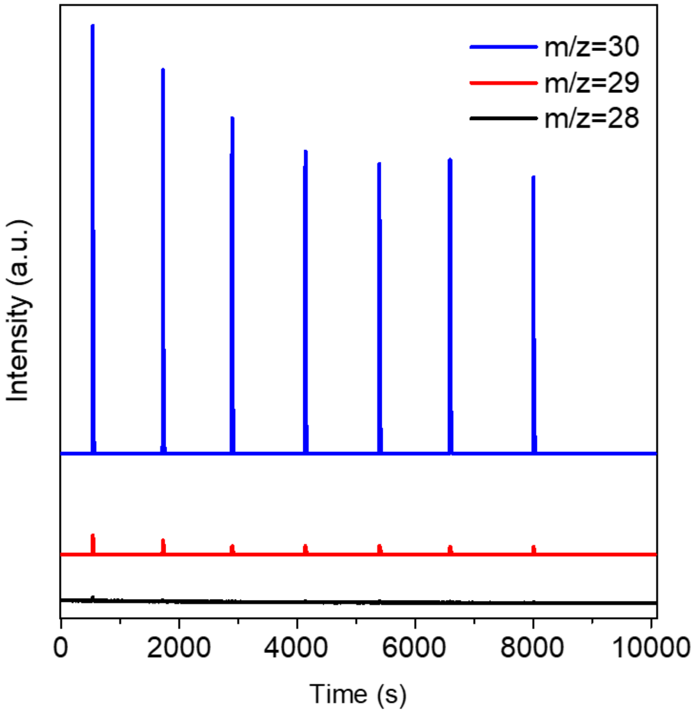

Figure S14. (a) Time evolution of mass spectra peaks at m/z=28, m/z=29, and m/z=30 during pure $^{15}N_2$ (20 kPa) treatment of Ca_2N at 400°C. (b) Intensity of peaks at m/z=28, 29, and 30 monitored as a function of the reaction time in panel a. The mass signal intensity of m/z=29 was not increased with reaction time, which means lattice nitrogen species did not attend the nitrogen dissociation process and exchange with molecular nitrogen ($^{15}N_2$).

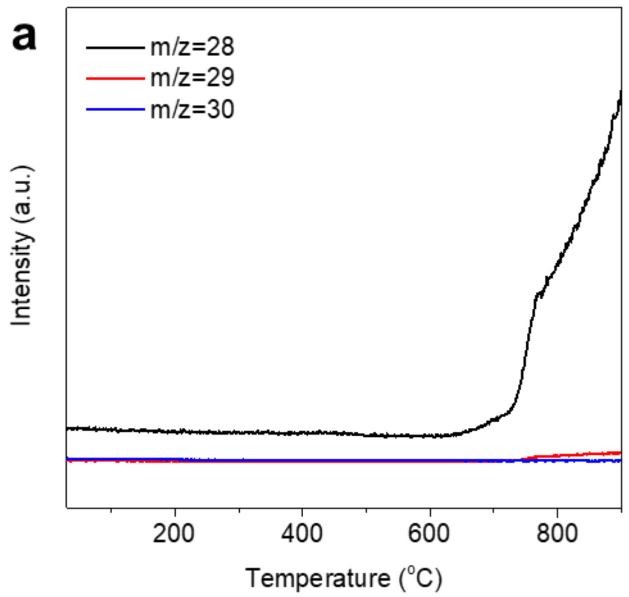

Figure S15. TPD for N_2 for Ca_2N collected by treatment with 20 kPa of pure $^{14}N_2$ (a) or $^{15}N_2$ at 400 °C for 2 h.

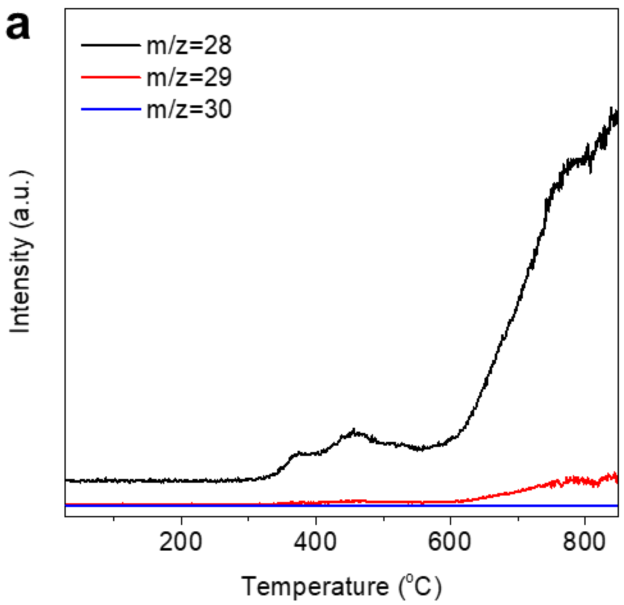

Figure S16. TPD for N_2 for Sr_2N collected by treatment with 20 kPa of pure $^{14}N_2$ (a) or $^{15}N_2$ at 400°C for 2 h.

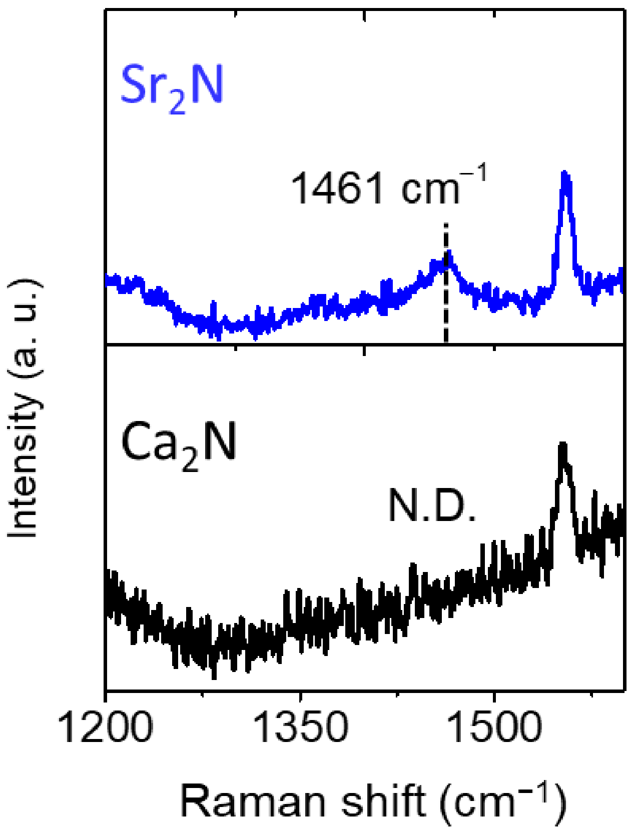

Figure S17. Raman spectra of Sr_2N (top) and Ca_2N (bottom).

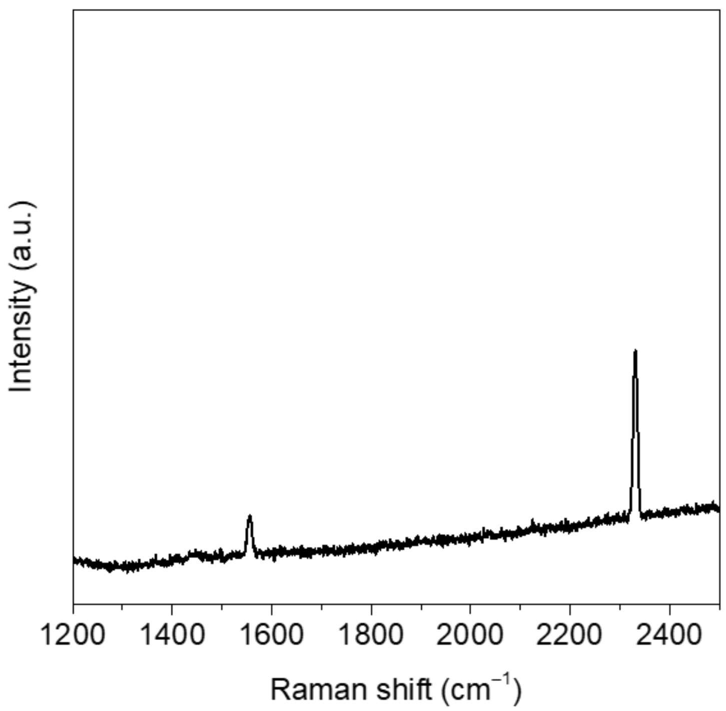

Figure S18. Raman spectra of Ba_2N pretreated in an Ar flow at 600°C for 2 h.

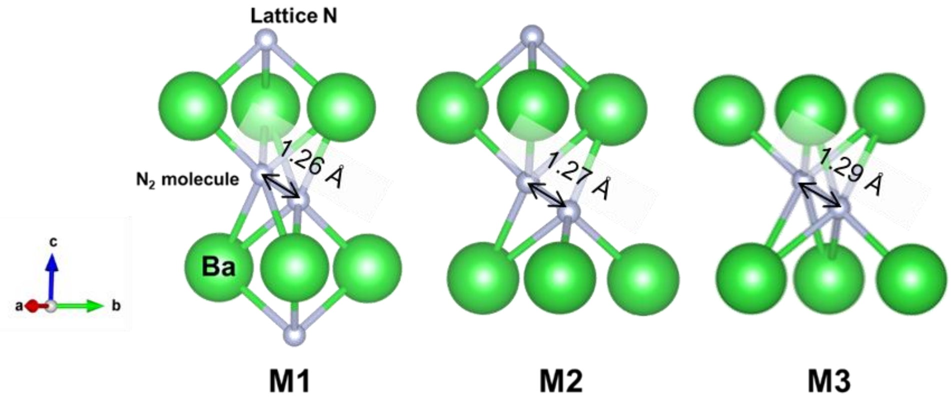

Figure S19. Local structure of N_2 absorbed on Ba_2N lattice.

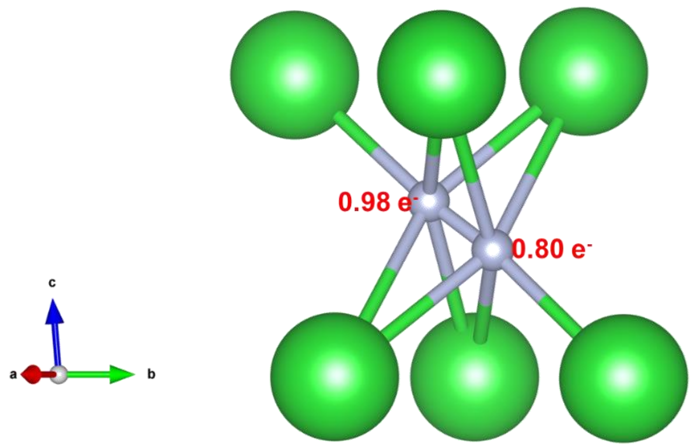

Figure S20. Bader charge analysis of one absorbed N_2 molecule in a model of $Ba_{24}N_{12}$+3N_2.

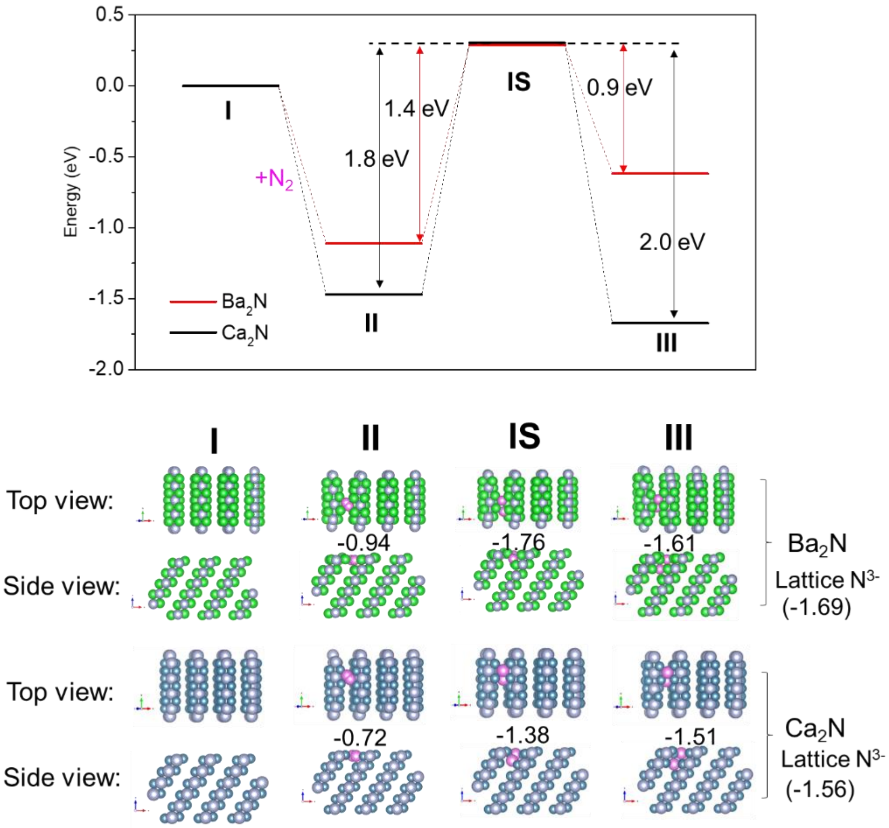

Figure S21. Calculated energy diagram for N_2 dissociation in interlayer space of Ba_2N and Ca_2N. The corresponding structure for N_2 dissociation on each material and Bader charge of nitrogen species in different active states are shown below the energy diagram.

Firstly, N_2 molecule incorporated into the interlayer space of Ba_2N is activated by the interlayer electrons to form $(N=N)^{2-}$ that sandwiched by 2 cationic slabs of $[Ba_2N]^+$ as the main intermediate (State I→II). This process had been carefully determined based on both experimental and theoretical results. Then, the N_2 is dissociated to form N^{3-} (determined by Badar charge analysis) with an energy barrier of 1.4 eV (State II→III). In contrast, the N_2 dissociation energy barrier for Ca_2N is 1.8 eV, which is larger than that (1.4 eV) of Ba_2N. This difference is due to that electron transfer from Ba_2N to N_2 molecule effectively takes place than the case of Ca_2N since the work function of Ba_2N is much smaller than that of Ca_2N (Figure S22). This calculation agrees well with experimental results.

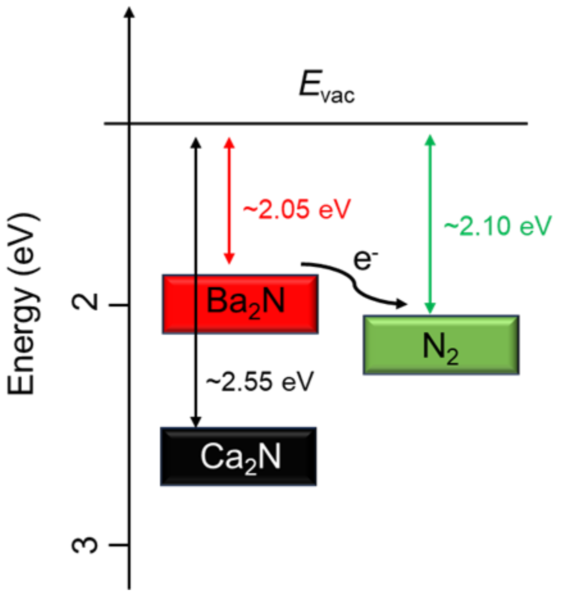

Figure S22. Schematic illustration of energy levels for Ba_2N, Ca_2N and N_2 molecule and transfer of charge from Ba_2N to N_2.

Table S1. Bond length and formation energy for different structures of N_2 incorporated in the interlayers of Ba_2N.

Model	Bond length [Å]	Formation energy [eV]
M1	1.26	–0.88
M2	1.27	–1.01
M3	1.29	–1.58

Table S2. Vibration frequencies and bond length for N_2 in the interlayers of Ba_2N with various concentrations of N_2 determined by DFT simulations.

**Model**	**Simplest formula**	**Bond length [angstrom]**	**Vibration frequencies [cm^{-1}]**	**Formation energy [eV]**
$Ba_{36}N_{18}$+N_2	$Ba_2N_{1.1111}$	1.29	1175	–1.57
$Ba_{36}N_{18}$+3N_2	$Ba_2N_{1.3333}$	1.26	1294, 1286, 1282	–3.68
$Ba_{24}N_{12}$+3N_2	$Ba_2N_{1.5}$	1.23	1448, 1432, 1424	–2.92

Table S3. Electron concentration for Ba_2N determined by iodine titration experiments.

Sample	Weight (mg)	e^- concentration (mol/g)	y
Ba_2N:ye^- (01)	16.9	0.00197411	0.613877416
Ba_2N:ye^- (02)	18.7	0.002116371	0.602097086
Ba_2N:ye^- (03)	20.6	0.002075758	0.572613061

References

(1) Kresse, G.; Furthmüller, J. Efficient iterative schemes for abinitio total-energy calculations using a plane-wave basis set. *Phys. Rev. B* **1996**, *54*, 11169−11186.

(2) Kresse, G.; Furthmüller, J. Efficiency of ab-initio total energy calculations for metals and semiconductors using a plane-wave basis set. *Comput. Mater. Sci*. **1996**, *6*, 15−50.

(3) Perdew, J. P.; Burke, K.; Ernzerhof, M.; Generalized Gradient Approximation Made Simple. *Phys. Rev. Lett.* **1996**, *77*, 3865–3868.

(4) Momma, K.; Izumi, F. VESTA 3 for three-dimensional visualization of crystal, volumetric and morphology data. *J. Appl. Crystallogr*. **2011**, *44*, 1272−1276.

(5) Tang, W.; Sanville, E.; Henkelman, G. *J. Phys.: Condens. Matter* **2009**, *21*, 4.

(6) Henkelman, G.; Uberuaga, B. P.; Jónsson, H. A climbing image nudged elastic band method for finding saddle points and minimum energy paths. *J. Chem. Phys.* **2000**, *113*, 9901– 9904.
